# Dose-Dependent Effects of Insulin-Like Growth Factor 1 in the Aged Olfactory Epithelium

**DOI:** 10.3389/fnagi.2018.00385

**Published:** 2018-11-20

**Authors:** Rumi Ueha, Kenji Kondo, Satoshi Ueha, Tatsuya Yamasoba

**Affiliations:** ^1^Department of Otolaryngology, The University of Tokyo, Tokyo, Japan; ^2^Division of Molecular Regulation of Inflammatory and Immune Diseases Research Institute for Biomedical Sciences, Tokyo University of Science, Chiba, Japan

**Keywords:** olfactory receptor neurons, aging, insulin-like growth factor 1, dose effects, proliferation, apoptosis

## Abstract

**Background:** Olfaction is known to be impaired by aging. We hypothesized that insulin-like growth factor-1 (IGF-1) administered at an appropriate dose could prevent age-induced negative effects on olfactory receptor neurons (ORNs). We explored the effects of low- and high-dose administration of IGF-1 on the ORN cell system in aged mice and investigated the involvement of the cellular mechanisms of IGF-1 in the regeneration of ORNs in aged mice.

**Methods:** We subcutaneously administered recombinant human IGF-1 (rhIGF-1) to 16-month-old male mice over 56 days, and then examined the histological effects of rhGF-1 on cellular composition, cell proliferation, and cell death in the aged olfactory epithelium (OE), by comparing among saline-treated and low- and high-dose rhIGF-1-treated mice.

**Results:** Low-dose rhIGF-1 administration increased the numbers of olfactory progenitors, immature ORNs, and mature ORNs in the OE, despite an increase in Cas3+ apoptotic cells. Notably, high-dose rhIGF-1 administration increased the numbers of only immature ORNs, not olfactory progenitors and mature ORNs, with a concurrent increase in apoptotic cells.

**Conclusion:** Our data suggest that in aged mice, IGF-1 administered at an appropriate dose could increase the number of mature ORNs and further human studies may contribute to the development of treatments for aging-related olfactory impairment.

## Introduction

Olfaction is known to be impaired by aging. Aging negatively affects the ORN cell system in the olfactory neuroepithelium (OE) (Ueha et al., 2018a). The OE has a unique regenerative stem-cell system, which is maintained by the life-long replenishment of mature ORNs in the luminal layer from the progenitor cells in the basal layer (Costanzo, 1991; Schwob, 2002; Supplementary Figure S1). In steady-status neurogenesis, primitive olfactory progenitor cells differentiate into late progenitor cells and then differentiate into immature ORNs. A variety of stimuli, such as cytokines, neurotrophins, and growth factors, induce differentiation and maturation of immature ORNs (Buiakova et al., 1996; Nickell et al., 2012; Heron et al., 2013).

Neurotrophins and growth factors have important roles in neuronal differentiation, maturation, and maintenance in the steady-state condition (Fernandez and Torres-Aleman, 2012), and the dose-dependent differential effects of neurotrophins and growth factors have been reported (Tannemaat et al., 2008; Santos et al., 2016). Insulin-like growth factor-1 (IGF-1) is a growth factor that promotes neurogenesis and neuronal growth. Although IGF-1 is mainly produced in the liver and skeletal muscles, it is also generated in the central nervous system, where it plays an important role in the differentiation and maturation of neurons (Nishida et al., 2011; Nieto-Estevez et al., 2016; Pardo et al., 2016). As our previous study showed, reduction in ORN number and cell proliferation in the aged OE reduced gene expression of IGF-1, which may contribute to olfactory impairment during aging (Ueha et al., 2018a). Considering the previous studies, the balance of IGF-1 might be of importance in the regulation of OE homeostasis in the aging population.

We hypothesized that IGF-1 administered at an appropriate dose could restore the aging-induced reduction of ORNs. In the present study, we explored the effects of low-dose and high-dose IGF-1 administration on the ORN cell system in aged mice.

## Materials and Methods

### Mice

Sixteen-month-old male mice (*n* = 6 for each group) were used (C57BL/6, purchased from Saitama Experimental Animals, Saitama, Japan). The mice were maintained in a temperature-controlled (24 ± 1°C) environment under a 12-h light-dark cycle (light on from 09:00 to 21:00). Food and water were available *ad libitum*. All animal experiments were approved by the Animal Care and Use Committee of the University of Tokyo (Approval No. P17-010) and were conducted in accordance with institutional guidelines.

### Mouse Model of IGF-1 Administration

**FIGURE 1:**
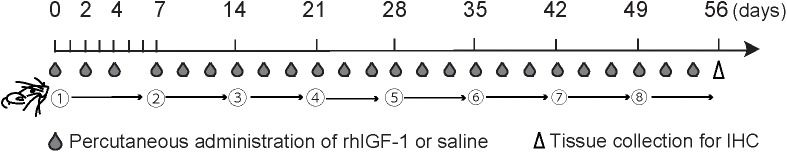
Experimental timeline. Mice were injected subcutaneously with 20 μg/kg (low-dose group) and 200 μg/kg (high-dose group) recombinant human insulin-like growth factor-1 (rhIGF-1) or saline three times a week from days 0 to 53. Subsequently, the olfactory epithelium was collected for analysis by immunohistochemistry (IHC).

Mice were injected subcutaneously with 20 μg/kg (low-dose group) and 200 μg/kg (high-dose group) rhIGF-1 (rhIGF-1; Mecasermin, Orphan Pacific, Tokyo, Japan), which is also active in mice (Castro et al., 2014; Khwaja et al., 2014), dissolved in sterile saline, three times a week from days 0 to 53 (Castro et al., 2014; Khwaja et al., 2014; Ueha et al., 2016b; Figure 1). Control mice received saline solution subcutaneously on the same schedule as the rhIGF-1-treated mice. Both control and rhIGF-1-treated mice were sacrificed on day 56.

### Tissue Preparation and Histological Analyses

The septal nasal mucosa was harvested and prepared for histological analyses as described previously (Ueha et al., 2014, 2016a,b, 2018a,b). In brief, all samples were cut at the level of the anterior end of the olfactory bulb. Four-micrometer-thick paraffin sections were deparaffinized and stained with hematoxylin and eosin or immunohistochemically. Antigen-retrieval and blocking of endogenous peroxidase activity were performed before immunostaining. Then, the sections were incubated in Blocking One (Nacalai Tesque, Kyoto, Japan) to block non-specific antibody binding. Primary antibodies against mouse SOX2 (SOX2; 1:300 dilution; rabbit monoclonal, Abcam clone EPR3131; Cambridge, United Kingdom), growth-associated protein 43 (GAP43; 1:1000 dilution; rabbit polyclonal, Novus #NB300-143B; Littleton, CO, United States), Ki-67 (1:200 dilution; rabbit monoclonal, Novus #NB600-1252), OMP (OMP; 1:8000 dilution, goat polyclonal, Wako, Osaka, Japan), and cleaved Cas3 (Cas3; 1:300 dilution; rabbit polyclonal, Cell Signaling #9661; Danvers, MA, United States) were detected with peroxidase-conjugated appropriate secondary antibodies and a diaminobenzidine substrate. In the OE, SOX2 is a transcription factor and expressed by proliferating progenitor cells in the basal layer. GAP43 and OMP are expressed by immature ORNs and mature ORNs, respectively. Ki-67 is a marker for cell proliferation and expressed in all active stages of the cell cycle, and Ki-67-positive cells are particularly detected in the basal layer (Ueha et al., 2018a,b). Caspases are crucial mediators of programmed cell death (apoptosis) and Cas3 is a well-defined activated death protease (Porter and Janicke, 1999).

Images of the bilateral septal OE were acquired using a digital microscope (BZ-X700; Keyence, Osaka, Japan) at 400× magnification. Using a previously reported method (Ueha et al., 2018a), the number of OMP^+^ ORNs, GAP43^+^ immature ORNs, and cleaved Cas3^+^ apoptotic cells per 1-mm length areas of the OE and the number of SOX2^+^ ORN progenitors and Ki-67^+^ cells on the basal layer per mm length of the OE were counted manually using digital imaging software (Photoshop CS6; Adobe, San Jose, CA, United States) in a blinded manner.

### Statistical Analysis

Statistical comparisons among groups were performed by one-way analysis of variance with Tukey *post hoc* tests using the GraphPad Prism software version 6.0 (GraphPad Software Inc., San Diego, CA, United States^1^). *P* < 0.05 was considered to be statistically significant.

## Results

**FIGURE 2:**
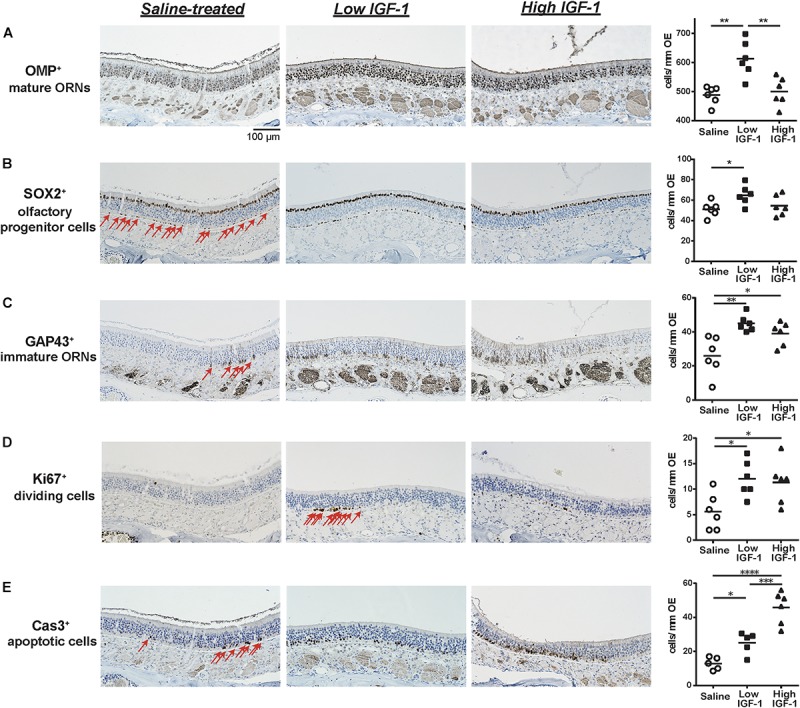
Representative images of immunohistological staining (brown) of OMP-positive (OMP^+^) cells **(A)**, SOX2^+^ ORN progenitor cells **(B)**, GAP43^+^ immature ORNs **(C)**, Ki67^+^ proliferating cells **(D)**, and cleaved Cas3^+^ apoptotic cells **(E)**. Each cell except for many OMP^+^ cells is indicated by arrows. Tissue sections were counterstained with the nuclear dye hematoxylin (blue). Numbers of SOX2^+^ ORN progenitors and Ki67^+^ actively proliferating cells per mm of the basal layer and OMP^+^ mature ORNs, GAP43^+^ immature ORNs, and Cas3^+^ apoptotic cells per mm of the OE in saline or rhIGF-1-treated mice. Open circles, rectangles, and triangles represent the values for each mouse in the saline, low-IGF-1, and high-IGF-1 treated groups (each *n* = 6), respectively. The horizontal lines represent the mean value for each group. ^∗^*P* < 0.05; ^∗∗^*P* < 0.01; ^∗∗∗^*P* < 0.001; and ^∗∗∗∗^*P* < 0.0001 (one-way ANOVA).

We first investigated the influence of rhIGF-1 on the cellular composition of the aged OE by comparing the OE of saline-and rhIGF-1-treated mice (low IGF-1, high IGF-1). We found that the number of OMP^+^ mature ORNs was significantly higher in the low-dose rhIGF-1-treated group compared to the saline-treated and high-dose rhIGF-1-treated groups. The number of SOX2^+^ olfactory progenitors also increased only in the low-dose rhIGF-1-treated group. However, the number of GAP43^+^ immature ORNs significantly increased in both the low- and high-dose rhIGF-1-treated groups compared to the saline-treated group (Figures 2A–C and Supplementary Figure S1).

We next examined the cellular mechanisms underlying the increase of mature and immature ORNs in rhIGF-1-treated mice. As the number of mature ORNs is determined by the balance between proliferation of the ORN precursors and cell death, we analyzed the number of Ki-67^+^ proliferating cells and Cas3^+^ apoptotic cells in the OE. Ki-67^+^ cells were mainly detected in and proximal to the basal layer, where olfactory progenitors and immature ORNs give rise to differentiated progenies. The number of Ki-67^+^ cells was significantly higher in both the low- and high-dose rhIGF-1-treated groups than in the saline-treated group. Interestingly, the number of Cas3^+^ apoptotic cells increased in the rhIGF-1-treated group compared to the saline-treated group, and this increase was more prominent in the high-dose rhIGF-1-treated group. Cas3^+^ cells were mainly detected in the basal and the intermediate layers, where immature ORNs are usually present (Figures 2D–E and Supplementary Table S1).

## Discussion

Here, we demonstrated, for the first time, that low-dose rhIGF-1 administration increased the number of OMP+ mature ORNs in the aged OE, but high-dose rhIGF-1 did not have positive effects on the aged OE. These dose-dependent effects of rhIGF-1 on the aged OE may be important for developing treatments for aging-related olfactory dysfunction.

IGF-1 is a growth factor exerting trophic effects on neuronal development and regeneration in the central nervous system and peripheral nervous system (Ishii et al., 1994; Bianchi et al., 2017). IGF-1 activates protein synthesis in neurons, glia, oligodendrocytes, and Schwann cells; stimulates the survival of mature and immature neurons, and promotes the proliferation of neuronal precursors (Devesa et al., 2011; Bianchi et al., 2017), acting neuroprotectively after injury. Thus, IGF-1 controls tissue homeostasis throughout the lifespan via regulation of cell proliferation, survival, and cell death (Kooijman, 2006). Recently, IGF-1 was found to have contradictory effects on the regulation of apoptosis. IGF-1 reportedly prevents the expression of apoptosis-associated genes and inhibits apoptotic programs and pathways (Parrizas et al., 1997; Kang et al., 2003; Fernandez et al., 2004; Vincent et al., 2004), but on the other hand, it is considered to induce apoptosis (Granerus et al., 2001; Raile et al., 2003; Saile et al., 2004; Fu et al., 2007). These different effects may be influenced by the dose, administration route, number of administrations, and the interval between administrations (Santos et al., 2016; Bianchi et al., 2017). IGF-1 secretion decreases and the effects of IGF-1 on tissues alter with aging (Obermayr et al., 2005; Apel et al., 2010; Bianchi et al., 2017). Therefore, it is reasonable to consider that IGF-1 plays an important role in the prevention and treatment of many neurological diseases such as olfactory and neurodegenerative disorders and hearing loss and IGF-1 therapies at an appropriate dose could provide significant benefits for aged individuals. How IGF-1 acts on OE homeostasis remains unclear. However, considering that IGF-1 and the IGF-1 receptor (IGF-1R) are expressed in the OE and the olfactory bulb (Scolnick et al., 2008; Lee et al., 2018) and IGF-1 is transported to the olfactory bulb via the olfactory nerve-related nasal transport (Shiga et al., 2014), systemic administration of IGF-1 may directly and indirectly influence the OE.

The increase in the number of OMP^+^ mature ORNs after IGF-1 administration can be partly explained by the overwhelmingly higher cell proliferation of olfactory progenitors and immature ORNs compared to the increase in Cas3^+^ apoptotic cells. However, it should be noted that different doses could lead to different effects. We elucidated that high-dose rhIGF-1 administration increased the number of GAP43^+^ immature ORNs and accelerated apoptosis, but on the other hand, it did not cause an increase in the numbers of SOX2^+^ ORN progenitors and OMP^+^ mature ORNs. Collectively, these results indicate that the number of olfactory progenitors and immature and mature ORNs rises only by low-dose rhIGF-1 treatment and that high-dose rhIGF-1 administration did not increase the number of ORN progenitors and only promoted both proliferation and apoptosis of ORN progenitors/ immature ORNs in the aged OE. High-dose IGF-1 may saturate the IGF-1R, and free IGF-1 and the saturated IGF-1 – IGF-1R complexes could lead to an increase of apoptosis of ORN progenitors/ immature ORNs. Increased apoptosis is likely to be at least partly responsible for the unchanged ORN numbers in the high-dose rhIGF-1-treated mice.

Considering that the reduction in mature ORN numbers is associated with olfactory impairment in the aged population (Ueha et al., 2018b), IGF-1 administered at an appropriate dose could prevent aging-induced negative effects on ORNs. However, because our results were only obtained from mouse studies, care should be taken regarding IGF-1 application for the treatment of human olfactory impairment. Further studies are required to investigate the application of IGF-1 treatment to humans and to determine the appropriate dose of adequate intervals between IGF-1 administrations for the treatment of olfactory deterioration in aged individuals, and to assess the effects of IGF-1 administration on wellness and quality of life.

Low-dose, but not high-dose, rhIGF-1 administration increased the number of OMP+ mature ORNs in the aged OE. IGF-1 administration at an appropriate dose may be a key factor in treatments for aging-related olfactory dysfunction.

## Data Availability

The data that support the findings of this study are available from the corresponding author, RU, upon reasonable request.

## Author Contributions

RU, KK, and SU developed the concept, designed and performed the experiments, and analyzed the data. TY developed the concept and designed the experiments. All authors contributed to interpretation of the data and writing of the manuscript.

## Conflict of Interest Statement

The authors declare that the research was conducted in the absence of any commercial or financial relationships that could be construed as a potential conflict of interest.

## Footnotes

1 www.graphpad.com

## Supplementary Material

FIGURE S1 **A,B**: Representative images of the olfactory epithelium (OE) from untreated mice (hematoxylin and eosin stain; **A**, 40× magnification; **B**, 400× magnification). The box in **A** indicates the region of the OE shown in **B**,**C**: Diagram of cell lineage of olfactory receptor neurons (ORN).

TABLE S1 Change rate of each cell number. Each change rate was calculated by dividing each cell number in the low and high-dose insulin-like growth factor-1 (IGF-1) groups by those in the saline-treated control group. ^∗^*P* < 0.05; ^∗∗^*P* < 0.01; and ^∗∗∗∗^*P* < 0.0001 (one-way ANOVA). Cas3, caspase-3; GAP43, growth associated protein 43; OMP, olfactory marker protein; ORN, olfactory receptor neuron; SOX2, sex determining region Y-box 2.

## Abbreviations

Cas3: caspase-3
GAP43: growth associated protein 43
IGF-1: insulin-like growth factor-1
IGF-1R: insulin-like growth factor-1 receptor
OE: olfactory epithelium
OMP: olfactory marker protein
ORN: olfactory receptor neuron
rhIGF-1: recombinant human IGF-1
SOX2: sex determining region Y-box 2
