# Supplementary figures and images for: Dose-Dependent Effects of Insulin-Like Growth Factor 1 in the Aged Olfactory Epithelium
Source: Front Aging Neurosci. 2018 Nov 20;10:385. doi: 10.3389/fnagi.2018.00385 (PMC6256067; doi:10.3389/fnagi.2018.00385)

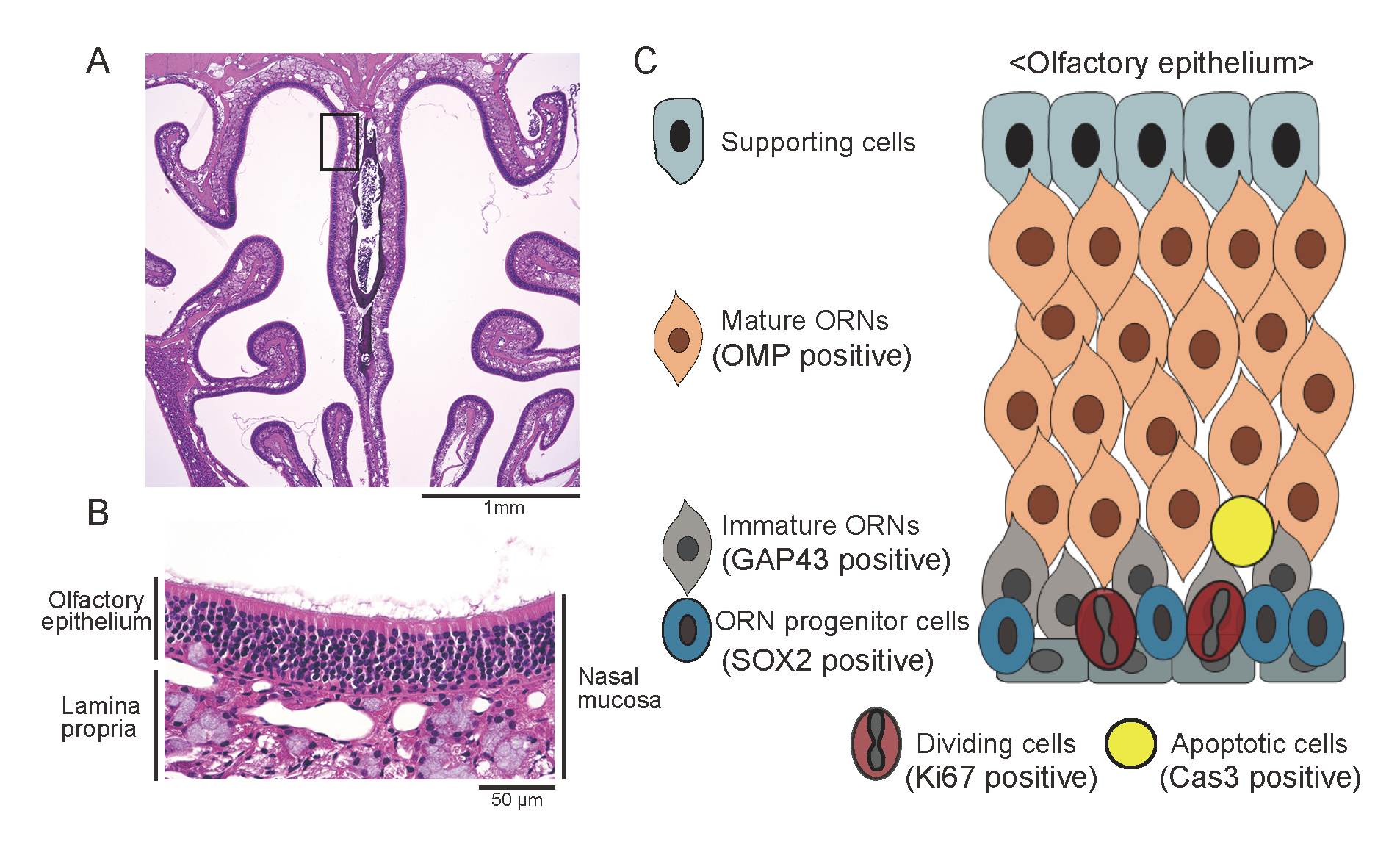

Supplement: FIGURE S1 — A,B: Representative images of the olfactory epithelium (OE) from untreated mice (hematoxylin and eosin stain; A, 40× magnification; B, 400× magnification). The box in A indicates the region of the OE shown in B,C: Diagram of cell lineage of olfactory receptor neurons (ORN). [file Image_1.TIFF]
